# Early changes in gene expression profiles in AML patients during induction chemotherapy

**DOI:** 10.1186/s12864-022-08960-4

**Published:** 2022-11-14

**Authors:** Ingrid Jakobsen, Max Sundkvist, Niclas Björn, Henrik Gréen, Kourosh Lotfi

**Affiliations:** 1 Division of Clinical Chemistry and Pharmacology, Department of Biomedical and Clinical Sciences, Faculty of Medicine and Health Sciences, Linköping University, Linköping, Sweden; 2 Department of Laboratory Medicine, School of Medical Sciences, Faculty of Medicine and Health, Örebro University, Örebro, Sweden; 3 Department of Forensic Genetics and Forensic Toxicology, National Board of Forensic Medicine, Linköping, Sweden; 4 Department of Hematology, Linköping University Hospital, 581 85 Linköping, Sweden

**Keywords:** AML, RNA sequencing, Gene expression, Time course analys

## Abstract

**Background:**

Elucidation of the genetic mechanisms underlying treatment response to standard induction chemotherapy in AML patients is warranted, in order to aid in risk-adapted treatment decisions as novel treatments are emerging. In this pilot study, we explored the treatment-induced expression patterns in a small cohort of AML patients by analyzing differential gene expression (DGE) over the first 2 days of induction chemotherapy.

**Methods:**

Blood samples were collected from ten AML patients at baseline (before treatment initiation) and during the first 2 days of treatment (Day 1; approximately 24 h, and Day 2; approximately 48 h after treatment initiation, respectively) and RNA was extracted for subsequent RNA sequencing. DGE between time points were assessed by pairwise analysis using the R package edgeR version 3.18.1 in all patients as well as in relation to treatment response (complete remission, CR, vs non-complete remission, nCR). Ingenuity Pathway Analysis (Qiagen) software was used for pathway analysis and visualization.

**Results:**

After initial data quality control, two patients were excluded from further analysis, resulting in a final cohort of eight patients with data from all three timepoints. DGE analysis demonstrated activation of pathways with genes directly or indirectly associated with NF-κB signaling. Significant activation of the NF-κB pathway was seen in 50% of the patients 2 days after treatment start, while iNOS pathway effects could be identified already after 1 day. nCR patients displayed activation of pathways associated with cell cycle progression, oncogenesis and anti-apoptotic behavior, including the STAT3 pathway and Salvage pathways of pyrimidine ribonucleotides. Notably, a significant induction of cytidine deaminase, an enzyme responsible for the deamination of Ara-C, could be observed between baseline and Day 2 in the nCR patients but not in patients achieving CR.

**Conclusions:**

In conclusion, we show that time-course analysis of gene expression represents a feasible approach to identify relevant pathways affected by standard induction chemotherapy in AML patients. This poses as a potential method for elucidating new drug targets and biomarkers for categorizing disease aggressiveness and evaluating treatment response. However, more studies on larger cohorts are warranted to elucidate the transcriptional basis for drug response.

**Supplementary Information:**

The online version contains supplementary material available at 10.1186/s12864-022-08960-4.

## Background

The standard induction chemotherapy in acute myeloid leukemia (AML) have remained roughly the same over the last decades, with treatment consisting of the nucleoside analog cytarabine (Ara-C) in combination with an anthracycline, most commonly daunorubicin or idarubicin. Additional drugs have been used together with the cornerstone drugs in different clinical trials, and in specific cases such as that of acute promyelocytic leukemia, treatments targeting specific chromosomal rearrangements are available [1]. However, AML patients still suffer from a significant degree of therapy-related mortality and treatment failure. More knowledge is needed of the genetic mechanisms underlying poor treatment response in AML, in order to achieve better risk stratification and individual adaption of treatment regimens as newer drugs are emerging in clinical trials. Genetic variation in genes encoding drug-metabolizing enzymes, transporters, DNA-repair systems or in genes regulating cell cycle progression and apoptosis is likely influencing cellular drug response both in leukemic and non-leukemic cells, although the clinical utility of such markers in relation to AML treatment decisions remain controversial [2]. In leukemia, gene expression profiling of diagnostic samples has been implicated as a tool able to discriminate between different types (e.g ALL and AML) [3] as well as molecular and prognostic subclasses of AML [4–6]. It has also been shown that acute lymphoblastic leukemia cells of different molecular subtypes share common but treatment-specific acute gene expression changes induced by mono- or combination therapy, as measured by gene expression arrays of samples collected before and 1 day after treatment [7]. In addition, a recent study of normal p53-regulated apoptosis, induced by DNA damage, demonstrated that the extent of the apoptotic response was attributable to the rapidity of the transcriptional response rather than to a specific set of target genes [8]. This shows that time series analysis of gene expression could provide new insights to the mechanisms behind drug response. The early drug-induced gene expression changes in AML patients undergoing standard induction chemotherapy has, to our knowledge, not been investigated previously. In this pilot study, we aimed to explore the expression patterns in a small cohort of AML patients and analyzing differential gene expression over the first 2 days of induction chemotherapy. Using RNA sequencing data, the changes in gene expression over time was analyzed overall to identify common patterns, but also compared between patients achieving complete remission and those who did not, to identify potential genes and pathways that might be used to predict or understand the response.

## Materials and methods

### Patients and sample collection

Ten patients with AML scheduled for induction chemotherapy at the hematology clinic of Linköping University Hospital were included in the study. The study was performed in compliance with the Declaration of Helsinki under the approval by the local ethics committee (Linköping, Sweden), and all patients gave informed consent. Patient characteristics and treatment details are summarized in Table 1. Blood samples were collected at baseline (before treatment initiation) and during the first 2 days of treatment (Day 1; approximately 24 h after treatment initiation, and Day 2; approximately 48 h after treatment initiation). All patients received Ara-C either as 100 mg/m^2^ as a continuous infusion (Patient 2) and Patient 3) or as 1 g/m^2^ twice daily as 2 h infusions before the Day 1 sample collection; before the Day 2 sample collection all patients except Patient 2 and Patient 3 had also received anthracycline treatment either as idarubicin (10 mg/m^2^ as 1 h infusion; Patient 1 and Patient 4) or daunorubicin (60 mg/m^2^ as 8 h infusion). Patient 4 was also part of a randomized phase 2 trial investigating the addition of cladribine, as a 5 mg/m^2^ 1 h infusion two times, to Ara-C and idarubicin [9]. Patient 5 was initially suspected to have acute promyelocyte leukemia and received treatment with all-trans retinoic acid (ATRA) before the final AML diagnosis and inclusion in this study. No cases of secondary AML were included. In addition to chemotherapy, the patients received standard supportive care with common co-medications including dexamethasone eye drops, allopurinol and antiemetics. RNA was extracted from EDTA blood samples using the Qiagen Blood RNA mini kit (Qiagen), or from blood samples stabilized in PAXgene blood RNA tubes (PreAnalytiX) using the PAXgene blood RNA kit (PreAnalytiX), according to the manufacturers´ instructions. The RNA concentration was measured on a NanoDrop (Thermo Scientific) and the RNA integrity number (RIN) was assessed using an Agilent 2100 BioAnalyzer (Agilent) with the Agilent RNA 6000 nano kit (Agilent) according to the manufacturer’s instructions.

**Table 1:** AML patient characteristics

Patient ID	Year of diagnosis	Age	Induction treatment regime	Blast count at baseline	Leukocyte paticle count at baseline (× 10^**9**^/L)	Leukocyte particle count Day 2 (48 h) (× 10^**9**^/L)	Cytogenetics	***FLT3***- ITD	***NPM1*** mutation	***CEBPA*** mutation	Morphological complete remission after induction^**c**^
1	2001	67	Idarubicin and cytarabine	80% (BM), 60% (PB)	60,8	58	Aberrant expression of T-cell markers	NA	NA	NA	No
2	2001	81	Daunorubicin and cytarabine^a^	NA	93	75,9	NA	NA	NA	NA	Yes
3	2003	39	Daunorubicin and cytarabine^a^	20% (PB)	51,7	31,6	Normal karyotype	No	No	NA	Yes
4	2001	67	Cladribine, cytarabine and idarubicin	90% (BM)	72	1,9*	Trisomy 8	NA	NA	NA	Yes
5	2003	66	Daunorubicin and low-dose cytarabine^b^	40% (BM)	35,8	11,2	1-40dmin [22] /46XY [3]	Yes	No	NA	No
6	2015	64	Daunorubicin and cytarabine	78% (BM), 57% (PB)	1,6	0,7	Normal karyotype	No	Yes	Heterozygous	Yes
7	2015	74	Daunorubicin and cytarabine	88% (BM)	164	2,6	Not possible due to no mitoses after culturing	NA	NA	NA	No
8	2015	73	Daunorubicin and cytarabine	60% (BM), 77% (PB)	135	37	Normal karyotype	Yes	No	No	No
9	2016	21	Daunorubicin and cytarabine	75% (BM), 44% (PB)	68	5,2	47XY, inv.(16)(p13;q22), +mar [23]	No	No	NA	Yes
10	2016	46	Daunorubicin and cytarabine	32% (BM), 2% (PB)	7,4	3,4	Complex karyotype	No	No	No	No

^a^Initially only cytarabine; no daunorubicin during study sample collection

^b^After one previous day of all-trans-retinoic acid (ATRA) treatment

^c^Bone marrow evaluated at day 15–28 after the start of induction chemotherapy

*Leukocyte particle count available also from Day 1 (24 h); 32*10^9^/L

### Sequencing and data preprocessing

All samples with a RIN > 7.5 were sent to the National Genomics Infrastructure (NGI, Stockholm, Sweden) hosted by the Science for Life Laboratory, for RNA sequencing. Libraries were prepared with the TruSeq RNA Library Prep Kit v2 (Illumina) with poly-A selection and paired-end (2x100bp) sequencing was performed on the NovaSeq 6000 Sequencing System (Illumina) using an s1 flow cell. Two samples, Patient 6 Day 2 and Patient 10 Day 2, failed library preparation and due to the low amount of extracted RNA no further attempts could be made. Preprocessing of the raw sequencing data was performed by NGI according to the nf-core/rnaseq pipeline (see https://github.com/nf-core/rnaseq for details). Briefly, the pipeline processes the raw fastq data using FastQC for read quality control and TrimGalore! for adapter removal and quality trimming. Alignment of the reads to the human reference genome GRCh38 was made using STAR version 2.7.2b [10]. The read distributions over genomic features were summarized using featureCounts from the Subread package version 1.6.2 [11, 12], and additional quality-control of the results was performed using DupRadar [13] for duplication rate quality control, edgeR [14, 15] for sample similarity, Preseq [16] for library complexity estimation, and RSeQC [17] for read distribution, duplication and gene body coverage. Finally, the quality measures were summarized using MultiQC version 1.7 [18].

### Differential gene expression analysis

To explore changes in gene expression in response to treatment, R version 3.6.1 [19] and the R package edgeR version 3.18.1 [14, 15] was used. The data was structured to contain read counts, ensemble gene ID, patient ID and sample time point, for all genes with read counts > 1 count per million (CPM) in ≥ 3 samples. The data was then normalized using the calcNormFactors to scale the raw library sizes for each sample. Differential gene expression (DGE) compared to baseline levels were investigated by pairwise comparisons. In the analysis of DGE between time points, were replicates were lacking for individual patients, the biological coefficient of variation (BCV) was defined as 0.4 as recommended in the edgeR user guide. Negative binomial generalized linear models (GLMs) were fitted for each patient using the function GLMFit with the dispersion defined as the BCV squared. Likelihood ratio tests were then performed with the function glmLRT to determine which genes were differentially expressed between the time points. For the overall analysis, the function estimateDisp was used to determine the trended, tagwise and common dispersion for the samples. These were then fitted using glmQLFit and a quasi-likelihood F-test was performed using glmQLFTest. Gene expression changes were also investigated in the clinical response groups, morphological complete remission (CR; final *n* = 4) and non-complete remission (nCR; final *n* = 4) as assessed by bone marrow sampling between day 15 and day 28 after treatment initiation.

### Pathway analysis

The function TopTags in edgeR was used to extract differentially expressed (DE) genes and their log_2_(fold change), *p*-value and false discovery rate adjusted *p*-value. DE genes with *p*-values ≤0.05 were used as input for Ingenuity Pathway Analysis (IPA) (QIAGEN) software that analyses and visualizes DE genes to identify activated or inhibited pathways [20]. Canonical pathways extracted from IPA with a − log(*p*-value) ≥ 1.3 (corresponding to a *p*-value < 0.05) were marked as inhibited if the Z-score was ≤ − 2 and activated if it was ≥2. All pathway images in the manuscript were generated using **IPA** (QIAGEN). Molecules are represented as nodes, and the biological relationship between two nodes is represented as an edge (line). All edges are supported by at least one literature reference or canonical information stored in the IPA knowledge base. The intensity of the node color indicates the degree of up- or down-regulation in red and green, respectively. Nodes are displayed using various shapes that represent the functional class of the gene product. Edges are displayed with various labels that describe the nature of the relationship between the nodes (e.g., P for phosphorylation, T for transcription).

## Results

### Sequencing data quality assessment

Based on high duplication rate, skewed GC distribution and large portion of multimapping reads, Patient 6 (baseline and 24 h samples) and Patient 10 (baseline and 24 h samples) were excluded from further analysis. The duplication rates in Patient 6 and 10 were 82,9% and 66,8% (at baseline) and 71,9% and 61,2% (at 24 h), respectively. For the remaining eight samples the overall duplication rate was 46,1 ± 2,48%; at baseline it was 47,4 ± 5,23%, at 24 h 45,2 ± 3,84% and at 48 h 45,6 ± 3,42% (mean ± 95%CI). The skewed GC distribution (see Supplementary Fig. 1) and high levels of overrepresented sequences (see Supplementary Fig. 2) are indicative of ribosomal, bacterial or viral RNA contamination, or biased selection during sample preparation. It could be noted that both these patients had extremely high expression of one single gene – *HBB* – encoding the hemoglobin subunit β-hemoglobin, with feature counts of several million. However, due to the overall quality metrics divergences, the decision to exclude these two patients remained. This resulted in a final cohort of eight patients with data from all three time points available. Summary statistics from MultiQC are shown in Additional file 3.

### Common changes in gene expression

**Fig. 1:**
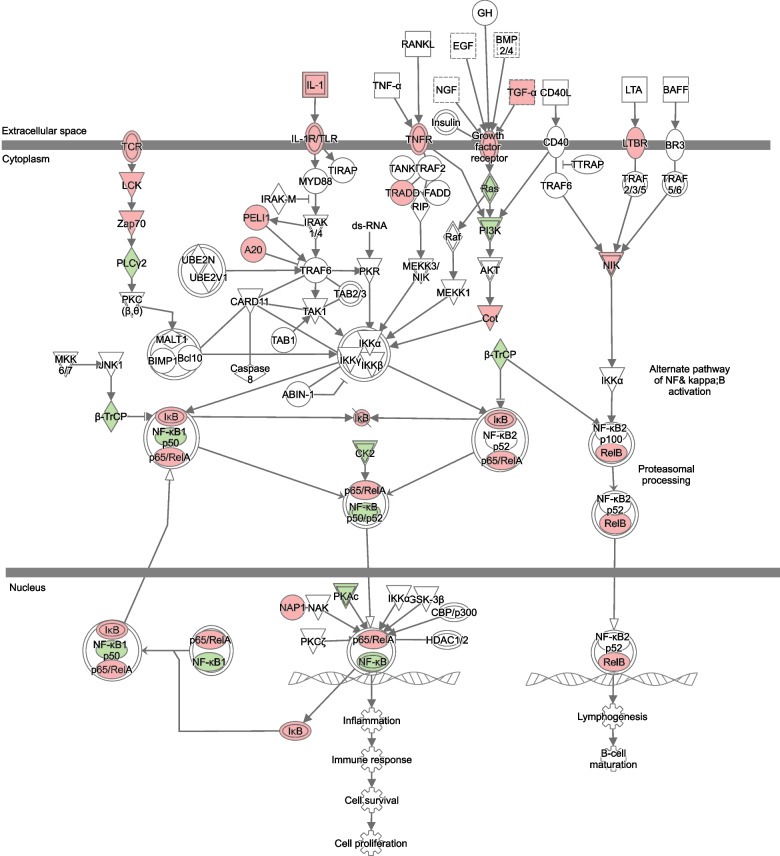
Activation of the NF-κB pathway. Genes marked in red are up-regulated between baseline and Day 2 while those marked in green are down-regulated

**Fig. 2:**
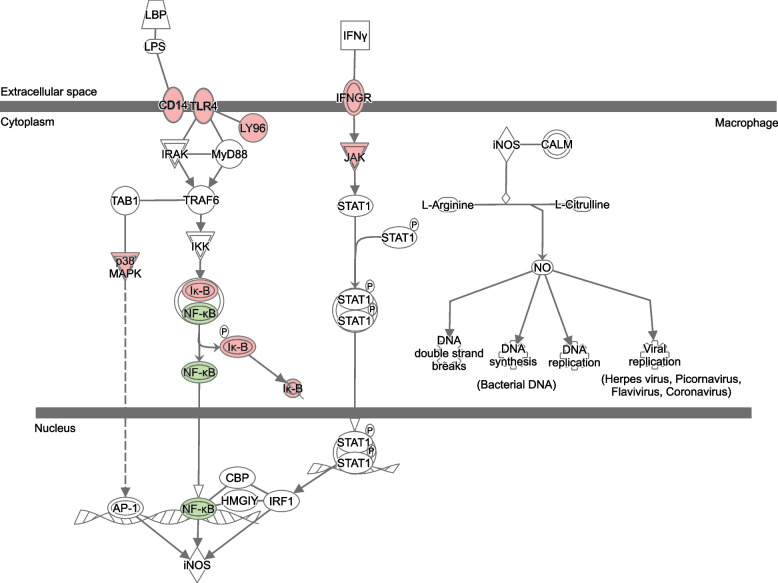
Activation of the iNOS pathway. Genes marked in red are upregulated between baseline and Day 2 while those marked in green are downregulated

Pairwise comparisons with the assessment of overlap between individual patients, and analysis of DGE overall between the time points of the whole cohort demonstrated significant activation of pathways with genes directly or indirectly associated with NF-κB signaling. This includes processes regulating immune responses, including inflammatory processes, proteasomal processing, lymphogenesis and cell survival as well as proliferation [21]. In addition, iNOS production is associated with NF-κB signaling, and this could be observed in our data set as DGE between time points in the iNOS pathway (Figs. 1 and 2 illustrate the significant transcriptional changes observed in the NF- κB pathway and iNOS pathway, respectively). The entire list of affected pathways with Z-scores can be found in Additional file 1, Table S1 (individual patient data) and Additional file 2, Table S2 (response groups and Overall data). Significant activation of the NF-κB pathway was seen in 50% of the patients (patient 1, 4, 5 and 7) 2 days after treatment start, while iNOS pathway effects could be identified already after 1 day; however this effect was evident only in the grouped analysis.

### Pathways affected in relation to treament outcome

**Fig. 3:**
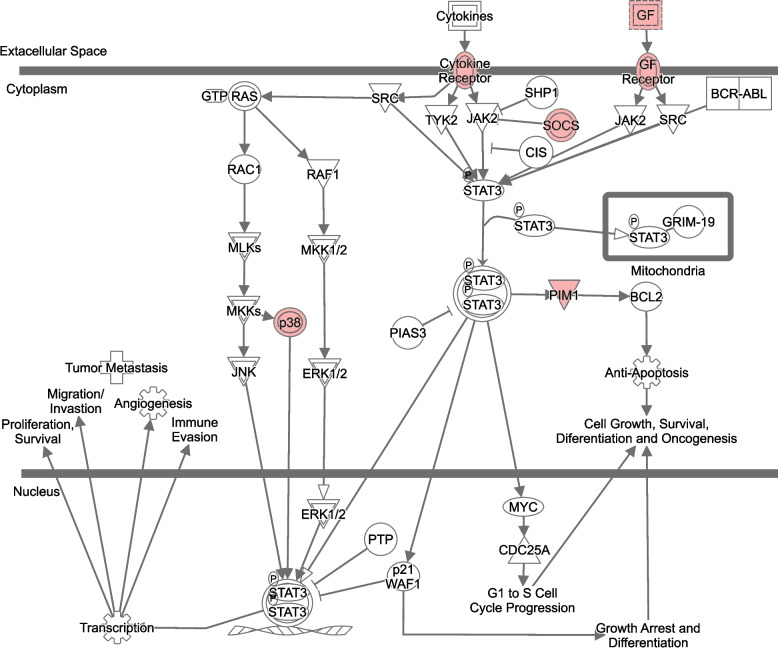
The STAT3 pathway. Genes marked in red are upregulated between baseline and Day 2, in patients that did not reach complete remission (CR) after the first course of induction chemotherapy

**Fig. 4:**
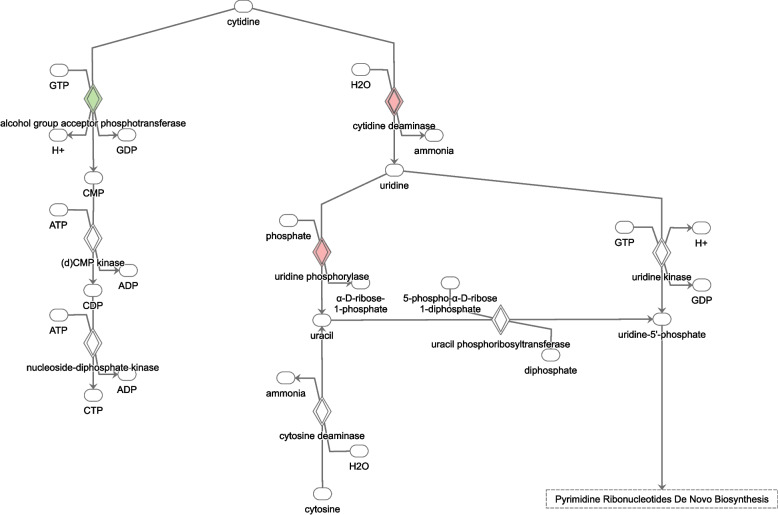
Salvage pathways of pyrimidine ribonucleotides. Genes marked in red are upregulated while those marked in green are downregulated between baseline and Day 2, in patients that did not achieve complete remission (nCR) after the first course of induction chemotherapy. Notably, increased expression of cytidine deaminase, responsible for the deamination of Ara-C into Ara-U, could be observed

We also investigated which pathways were affected during the treatment in relation to patient outcome; CR or nCR, based on day 15 or day 28 bone marrow evaluation. In patients reaching CR, the pathway Oxidative phosphorylation was activated already after the first treatment day, while the pathways Purine de novo biosynthesis II, tRNA charging, and Cyclins and cell cycle regulation were inhibited. These pathways were not significant in patients that did not reach CR. In contrast, nCR patients displayed activation of pathways associated with cell cycle progression, oncogenesis and anti-apoptotic behavior, including activation of the STAT3 pathway (Fig. 3) and Salvage pathways of pyrimidine ribonucleotides (Fig. 4). Notably, a significant induction of cytidine deaminase, an enzyme responsible for the deamination of Ara-C, could be observed between baseline and Day 2 in the nCR patients but not in the patients achieving CR. All pathways affected with their corresponding z-scores in the two response groups are listed in Additional file 2, Table S2, with selected relevant pathways summarized below in Tables 2 and 3.

**Table 2:** Pathways significantly affected in the CR patients

Pathway	24 h vs baseline	48 h vs 24 h	48 h vs baseline
Purine Nucleotides De Novo Biosynthesis II	–	Inhibited	Inhibited
tRNA Charging	–	Inhibited	Inhibited
Oxidative Phosphorylation	Activated	–	Activated
Cyclins and Cell Cycle Regulation	–	–	Inhibited

---**:** No significant difference

**Table 3:** Pathways significantly affected in the nCR patients

Pathway	24 h vs baseline	48 h vs 24 h	48 h vs baseline
STAT3 Pathway	Activated	Activated	Activated
Production of Nitric Oxide and Reactive Oxygen Species in Macrophages	Activated	Activated	Activated
IL-6 Signaling	Activated	Activated	Activated
Toll-like Receptor Signaling	Activated	Activated	Activated
Cell Cycle: G2/M DNA Damage Checkpoint Regulation	Activated	–	Activated
Salvage Pathways of Pyrimidine Ribonucleotides	–	Activated	Activated

---: No significant difference

## Discussion

In this pilot study, we analyzed gene expression changes over the first 2 days of standard induction chemotherapy in eight AML patients. We could show induction of relevant pathways related to immune response, cell proliferation and apoptosis, but also including genes of importance for drug pharmacokinetics. Some of these changes were detectable already after the first day of treatment. Overall, the NF-κB pathway was significantly activated after 2 days of treatment compared to baseline. This was also reflected by expression alterations in the iNOS pathway, which is directly linked to NF-κB signaling. Significant activation of the NF-κB pathway was seen in four patients 2 days after treatment start, while iNOS pathway effects could be identified already after 1 day; however this effect was evident only in the grouped analysis. This raises the question why we see this time delay between the activation of these two parallel pathways. The reason cannot easily be determined based on our present data. One possible explanation could be intrinsic relationships in the release and interactions between different cytokines and their respective downstream effects, such as IFNy preceeding the release of IL-1 and TGF-α. Due to the heterogeneous and limited number of samples in our study, further in-depth, high resolution studies of a larger number of samples would be needed in order to confirm and explore these specific time relationships.

The canonical NF-κB pathway is normally activated by pro-inflammatory cytokines, Toll-like receptor binding or T-cell receptor activation, subsequently leading to proteasomal degradation of the NF-κB inhibitor IκB. The release from IκB allows NF-κB to relocate from the cytoplasm to the nucleus where it regulates a wide variety of genes. In oncogenesis, this includes the transcription of genes promoting cell proliferation and survival as well as angiogenesis, metastasis, and suppression of the tumorsuppressors p53 and FOXO3a [21]. In AML, a growing body of evidence has demonstrated dysregulated NF-κB [22] and opened up the possibility of targeting this pathway; here, proteasome inhibitors are constituting a promising group of drugs with broad application to many types of cancers. In addition, in vitro experiments have shown that treatment with cytarabine induces NF-κB expression in the HL-60 cell line [23]. Our results in contrast show relative up-regulation of the IκB gene and correspondingly down-regulation of the NF-κB gene at 24 h (as part of the iNOS pathway; Fig. 2), and at 48 h (as part of the NF-κB pathway; Fig. 1) compared to baseline. This is indicative of an overall positive effect of the chemotherapy treatment, inhibiting cell proliferation and survival; however, we cannot determine whether these transcriptional changes are present in all cell populations (malignant as well as healthy) or to which extent these relative changes are reflecting a decreasing proportion of malignant cells (with assumed dysregulated NF-κB signaling). Interestingly, among the four patients with activation of the NFkB pathway as described above, three were non-responders after first induction (patient 1, 5 and 7) while patient 4 reached CR, as assessed by bone marrow sampling between day 15 and day 28 after treatment initiation. Patient 5 and 7 later reached morphological remission after additional treatment cycles; however patient 5 shortly thereafter relapsed with soft-tissue engagement of their AML, indicating residual disease after induction.

However, we should remember that this is a study on only eight patients.

When analyzing the patients based on their first response evaluation (bone marrow analysis at day 15 or 28), we were able to identify pathways that were significantly affected in one group but not the other. These differences could potentially be indicative of differences in tumor cell sensitivity to drug treatment. As such, the pathways Purine de novo biosynthesis II, tRNA charging, and Cyclins and cell cycle regulation were inhibited in patients reaching CR already after the first course of induction therapy, which might demonstrate reduced tumor cell proliferation in this group. These pathways were not significantly affected in the group of patients that did not achieve complete remission; however, two patients did so after additional treatment. In contrast to the CR group, nCR patients displayed significant activation of pathways associated with cell cycle progression, anti-apoptotic signaling and malignant transformation. Persistent activation of Stat3, as well as other Stat proteins, has been implicated as an oncogene promoting malignant transformation in various experimental settings [24, 25]. Stat3 target genes include Bcl-X and Bcl-2, both encoding anti-apoptotic proteins, and cooperation with c-Jun constitute yet another mechanism of interference with tumor cell apoptosis. In addition, the targets Cyclin D1 and Myc promote proliferation; in solid tumors an influence of Stat3 on VEGF expression could also further promote tumor growth and metastasis/tissue invasion through increased angiogenesis, a process of lower relevance for blood cancers. Many other target genes are likely involved in the oncogenic potential of Stat3, and activation of this protein does not necessarily lead to malignant transformation. However, suppression of Stat signaling has demonstrated tumor growth arrest, induction of apoptosis in cancer cells and inhibition of malignant transformation, indicating that it has potential as a future drug target [24, 26–28]. Our results suggest that patients displaying rapid activation of the Stat3 pathway could be harboring more resistant or highly proliferative leukemic clones. Interestingly, another pathway identified to be significantly activated in patients with poor initial treatment response was Salvage pathways of pyrimidine ribonucleotides. This pathway represents an important system for the maintenance of ribonucleotide pools in all proliferating cells, but also have implications for drug response [29, 30]. Notably, the induction of the enzyme cytidine deaminase (CDA) could result in a more treatment-resistant phenotype in these patients, as the pyrimidine analog Ara-C is subject to degradation into Ara-U by CDA. CDA induction as a mechanism of resistance to Ara-C has been demonstrated in vitro [31, 32], and our results support also an in vivo relevance.

Some limitations of this study need to be addressed and emphasize that the results needs to be kept in perspecitve. Our study population is small and the influence of interindividual variation may occlude common changes in gene expression related to the treatment, It should also be noted that these patient’s are heterogenous in their molecular diagnostics and treatment. We are partly compensating for this by comparing the different time point data to that from the baseline sample for each patient. However, the bulk RNA sequencing data represent the gene expression in both healthy and cancerous blood cells, and the leukemic blast proportion in blood samples from AML patients varies. Throughout the treatment, blood leukocyte counts are rapidly decreasing in many patients, and in our approach, we are not discriminating between cancer cells and healthy cells being eradicated. Changes in the relative abundance of different cell populations are likely to be reflected in the expression data, and unfortunately only leukocyte particle counts and not detailed peripheral blast counts for each sampling time point was documented in the patient journals. As an example, we cannot determine whether a decreased gene expression at day two compared to baseline means that the expression has been inhibited, or if it means that a specific cell population responsible for the baseline expression was eradicated, leaving only cell populations with another pattern. In addition, our patient samples were collected over a long period, and variations in treatment protocols and sampling procedures may have influenced the result. Initial principal component analysis indicated that the data tended to partly cluster depending on inclusion period (defined as before or after the introduction of national treatment guidelines, data not shown) although there was an overlap between these clusters.

In conclusion, we show that time-course analysis of gene expression represents a feasible approach to identify relevant pathways affected by standard induction chemotherapy in AML patients. This poses as a potential method for elucidating new drug targets and biomarkers for categorizing disease aggressiveness and evaluating treatment response. However, more studies on larger cohorts are warranted to elucidate the transcriptional basis for drug response, also assesing differences that are likely attributable to baseline characteristics such as cytogenetics and morphology. As single-cell RNA sequencing methodologies are developed and becoming more common, this would likely represent an option to further enhance the resolution also in time course studies like this one, although single-cell approaches are associated with other practical challenges in both sample management and analysis compared to conventional bulk RNA sequencing. Preferably, future studies would include more time points as well as follow-up analysis in the event of relapse.

## Supplementary Information

**Additional file 1.**

**Additional file 2.**

**Additional file 3.**

**Additional file 4.**

**Additional file 5.**

**Additional file 6.**

**Additional file 7.**

## Data Availability

The sequencing datasets generated and/or analysed during the current study are not publicly available due to restraints of the ethical permit, but are available from the corresponding author on reasonable request.
