# Supplementary material for: Early changes in gene expression profiles in AML patients during induction chemotherapy
Source: BMC Genomics. 2022 Nov 14;23:752. doi: 10.1186/s12864-022-08960-4 (PMC9664790; doi:10.1186/s12864-022-08960-4)
Supplement: Supplementary file 5 — Additional file 5. [file 12864_2022_8960_MOESM5_ESM.pdf]

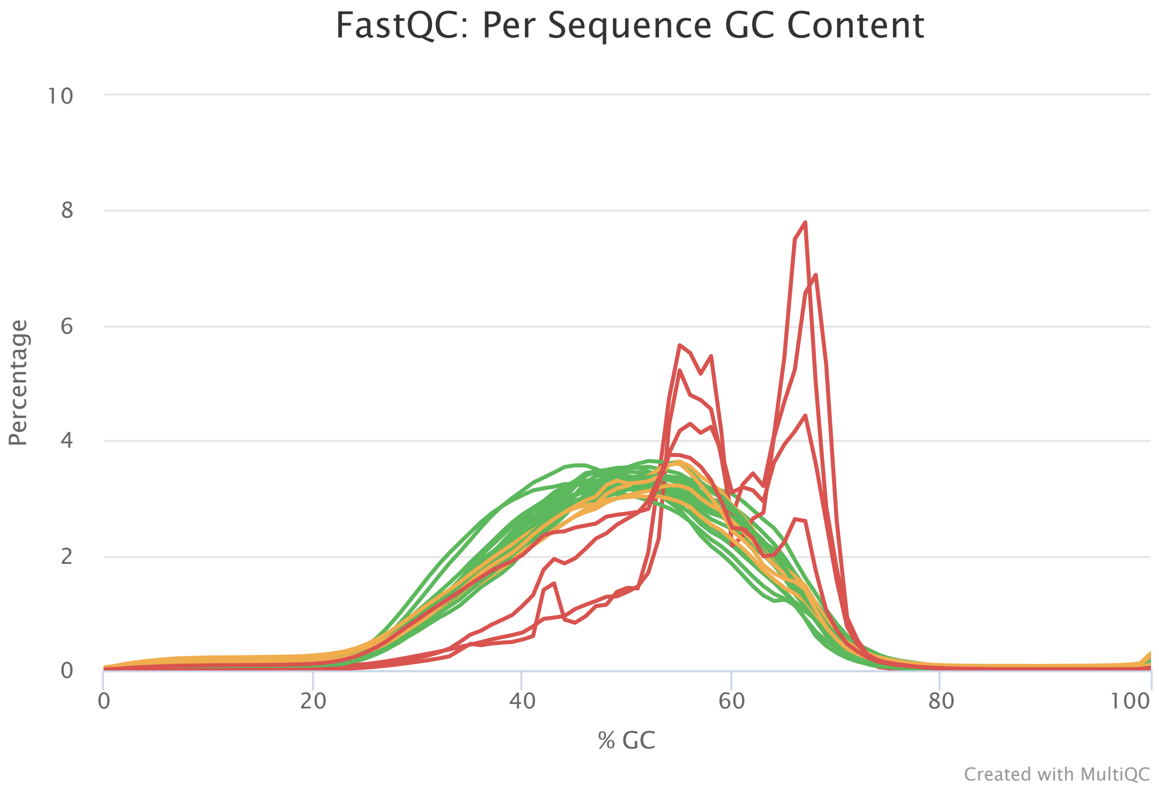


Supplementary Figure 1. Skewed GC distribution in samples from patient 6 and 10, corresponding to the red lines in the plot (fastQC data).
