# Supplementary material for: Early changes in gene expression profiles in AML patients during induction chemotherapy
Source: BMC Genomics. 2022 Nov 14;23:752. doi: 10.1186/s12864-022-08960-4 (PMC9664790; doi:10.1186/s12864-022-08960-4)
Supplement: Supplementary file 6 — Additional file 6. [file 12864_2022_8960_MOESM6_ESM.pdf]

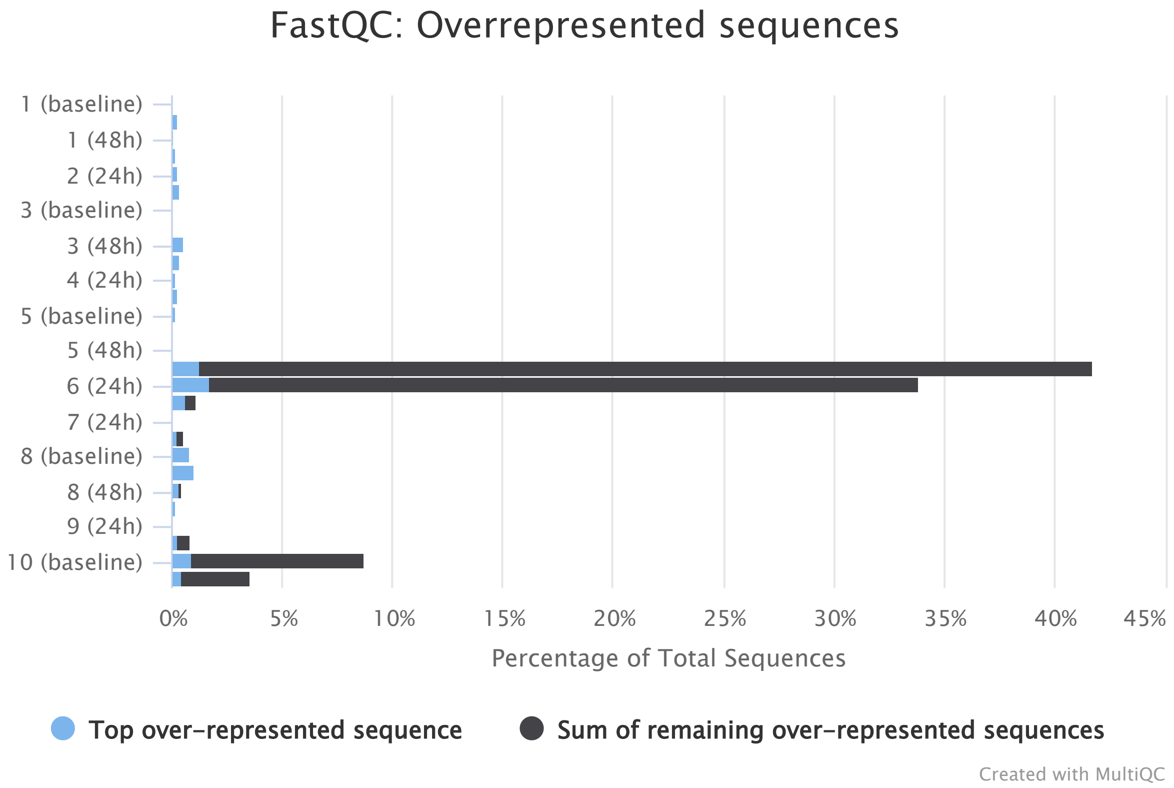

Supplementary Figure 2. High levels of overrepresented sequences in samples from patient 6 and 10 (fastQC data).
