# Supplementary material for: Early changes in gene expression profiles in AML patients during induction chemotherapy
Source: BMC Genomics. 2022 Nov 14;23:752. doi: 10.1186/s12864-022-08960-4 (PMC9664790; doi:10.1186/s12864-022-08960-4)
Supplement: Supplementary file 7 — Additional file 7. [file 12864_2022_8960_MOESM7_ESM.pdf]

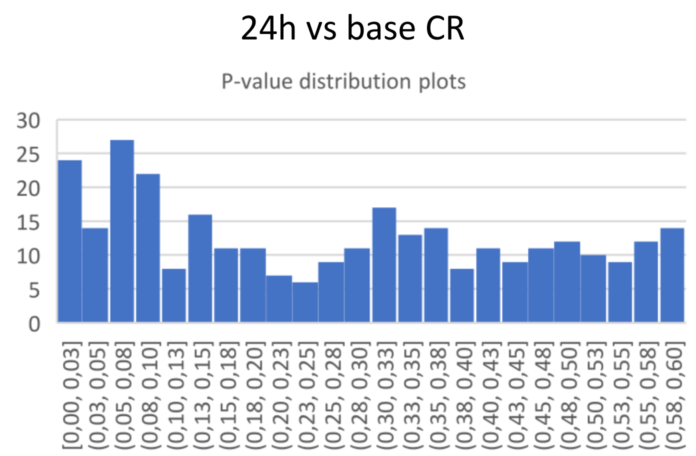
24h vs base CR
P-value distribution plots
30
25
20
15
10
5
0
[0,00, 0,03]
(0,03, 0,05]
(0,05, 0,08]
(0,08, 0,10]
(0,10, 0,13]
(0,13, 0,15]
(0,15, 0,18]
(0,18, 0,20]
(0,20, 0,23]
(0,23, 0,25]
(0,25, 0,28]
(0,28, 0,30]
(0,30, 0,33]
(0,33, 0,35]
(0,35, 0,38]
(0,38, 0,40]
(0,40, 0,43]
(0,43, 0,45]
(0,45, 0,48]
(0,48, 0,50]
(0,50, 0,53]
(0,53, 0,55]
(0,55, 0,58]
(0,58, 0,60]

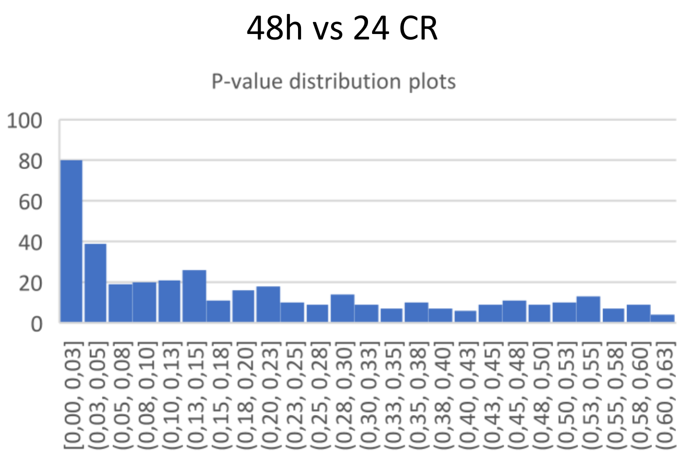
48h vs 24 CR
P-value distribution plots
100
80
60
40
20
0
[0,00, 0,03]
(0,03, 0,05]
(0,05, 0,08]
(0,08, 0,10]
(0,10, 0,13]
(0,13, 0,15]
(0,15, 0,18]
(0,18, 0,20]
(0,20, 0,23]
(0,23, 0,25]
(0,25, 0,28]
(0,28, 0,30]
(0,30, 0,33]
(0,33, 0,35]
(0,35, 0,38]
(0,38, 0,40]
(0,40, 0,43]
(0,43, 0,45]
(0,45, 0,48]
(0,48, 0,50]
(0,50, 0,53]
(0,53, 0,55]
(0,55, 0,58]
(0,58, 0,60]
(0,60, 0,63]

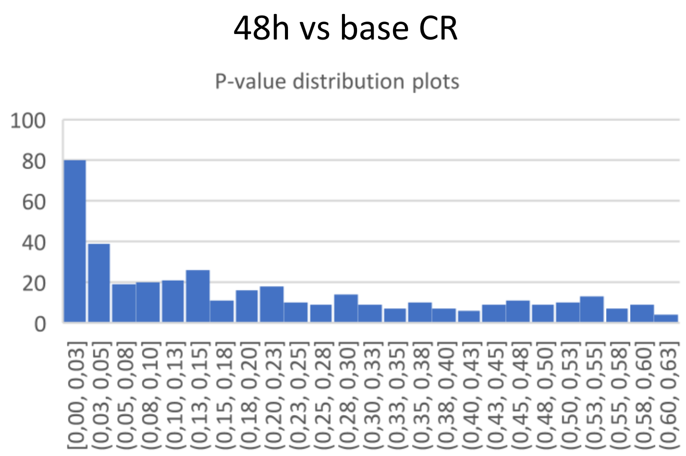
48h vs base CR
P-value distribution plots
100
80
60
40
20
0
[0,00, 0,03]
(0,03, 0,05]
(0,05, 0,08]
(0,08, 0,10]
(0,10, 0,13]
(0,13, 0,15]
(0,15, 0,18]
(0,18, 0,20]
(0,20, 0,23]
(0,23, 0,25]
(0,25, 0,28]
(0,28, 0,30]
(0,30, 0,33]
(0,33, 0,35]
(0,35, 0,38]
(0,38, 0,40]
(0,40, 0,43]
(0,43, 0,45]
(0,45, 0,48]
(0,48, 0,50]
(0,50, 0,53]
(0,53, 0,55]
(0,55, 0,58]
(0,58, 0,60]
(0,60, 0,63]

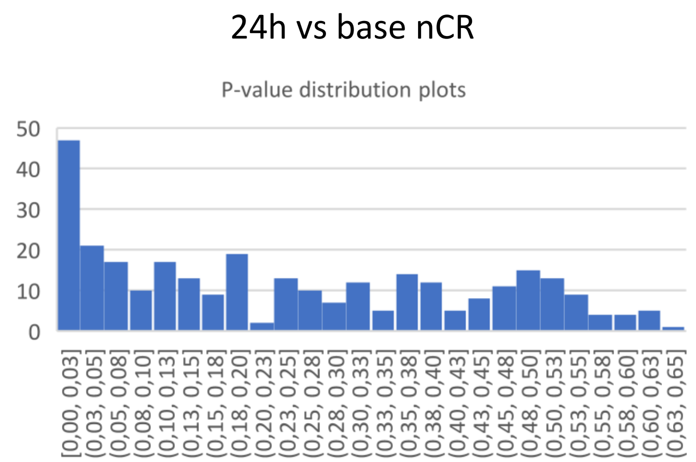
24h vs base nCR
P-value distribution plots
50
40
30
20
10
0
[0,00, 0,03]
(0,03, 0,05]
(0,05, 0,08]
(0,08, 0,10]
(0,10, 0,13]
(0,13, 0,15]
(0,15, 0,18]
(0,18, 0,20]
(0,20, 0,23]
(0,23, 0,25]
(0,25, 0,28]
(0,28, 0,30]
(0,30, 0,33]
(0,33, 0,35]
(0,35, 0,38]
(0,38, 0,40]
(0,40, 0,43]
(0,43, 0,45]
(0,45, 0,48]
(0,48, 0,50]
(0,50, 0,53]
(0,53, 0,55]
(0,55, 0,58]
(0,58, 0,60]
(0,60, 0,63]
(0,63, 0,65]

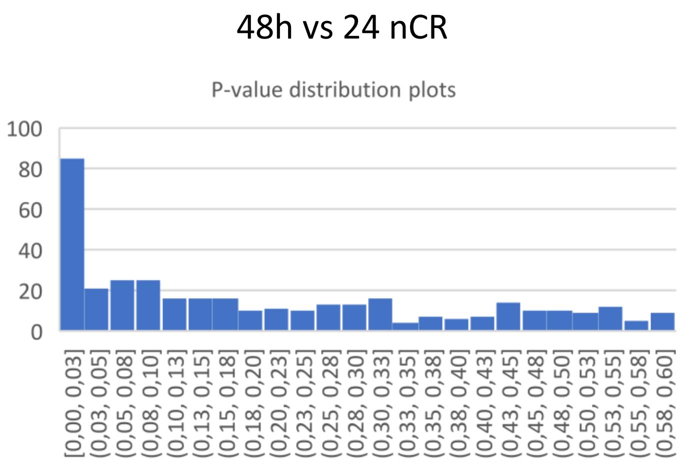
48h vs 24 nCR
P-value distribution plots
100
80
60
40
20
0
[0,00, 0,03]
(0,03, 0,05]
(0,05, 0,08]
(0,08, 0,10]
(0,10, 0,13]
(0,13, 0,15]
(0,15, 0,18]
(0,18, 0,20]
(0,20, 0,23]
(0,23, 0,25]
(0,25, 0,28]
(0,28, 0,30]
(0,30, 0,33]
(0,33, 0,35]
(0,35, 0,38]
(0,38, 0,40]
(0,40, 0,43]
(0,43, 0,45]
(0,45, 0,48]
(0,48, 0,50]
(0,50, 0,53]
(0,53, 0,55]
(0,55, 0,58]
(0,58, 0,60]

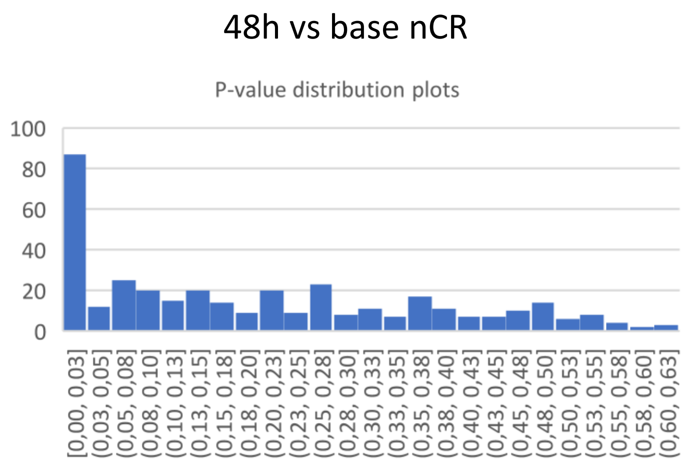
48h vs base nCR
P-value distribution plots
100
80
60
40
20
0
[0,00, 0,03]
(0,03, 0,05]
(0,05, 0,08]
(0,08, 0,10]
(0,10, 0,13]
(0,13, 0,15]
(0,15, 0,18]
(0,18, 0,20]
(0,20, 0,23]
(0,23, 0,25]
(0,25, 0,28]
(0,28, 0,30]
(0,30, 0,33]
(0,33, 0,35]
(0,35, 0,38]
(0,38, 0,40]
(0,40, 0,43]
(0,43, 0,45]
(0,45, 0,48]
(0,48, 0,50]
(0,50, 0,53]
(0,53, 0,55]
(0,55, 0,58]
(0,58, 0,60]
(0,60, 0,63]
